# Optimizing Surface Functionalization for Aptameric Graphene Nanosensors in Undiluted Physiological Media

**DOI:** 10.3390/s26020744

**Published:** 2026-01-22

**Authors:** Wenting Dai, Ziran Wang, Shifeng Yu, Kechun Wen, Yucheng Yang, Qiao Lin

**Affiliations:** Department of Mechanical Engineering, Columbia University, New York, NY 10027, USA; wd2319@columbia.edu (W.D.); wangziran@sdu.edu.cn (Z.W.); shifengy@cqu.edu.cn (S.Y.); kw2793@columbia.edu (K.W.); yy3280@columbia.edu (Y.Y.)

**Keywords:** biosensor, graphene field-effect transistor, aptamer, polyethylene glycol, C-reactive protein, undiluted physiological media

## Abstract

This paper presents the optimization of surface modification for aptameric graphene nanosensors for the measurement of biomarkers in undiluted physiological media. In these sensors, graphene transduces the binding between an aptamer and the intended target biomarker into a measurable signal while being coated with a polyethylene glycol (PEG) nanolayer to minimize nonspecific adsorption of matrix molecules. We perform a systematic study of the aptamer and PEG attachment schemes and parameters, including the impact of the serial or parallel PEG–aptamer attachment scheme, PEG molecular weight and surface density, and aptamer surface density on the sensor behavior, such as the responsivity to biomarker concentration changes, and importantly, they are used for operation in physiological media and have the ability to reject nonspecific binding to interfering molecules. We then use the understanding from this parametric study to identify graphene nanosensor designs that are optimally functionalized with PEG and aptamers to be strongly responsive to target biomarkers and effectively reduce nonspecific adsorption of interferents, thereby enabling sensitive and specific biomarker measurements in undiluted physiological media. The experimental results show that nanosensors that were optimized via serial modification with 5000 Da PEG at 15 mM and a 94 nt DNA aptamer at 500 nM allowed specific measurement of C-reactive protein (CRP) in undiluted human serum with a limit of detection (LOD) down to 27 pM, representing an up to 1000-fold improvement compared to previously reported CRP measurements.

## 1. Introduction

### Background

Affinity nanobiosensors detect and measure biomarkers based on their specific binding with affinity receptors such as antibodies [1,2], peptides [3,4], and aptamers [5,6]. Nanomaterials used in such devices have included, for example, silicon nanowires [7,8], carbon nanotubes [9,10], MXenes [11,12], transition metal dichalcogenides (TMDs) [13,14], and graphene [15,16], which have been demonstrated for a broad range of analytes such as ions [17,18], small molecules [19,20], and proteins [21,22]. Affinity nanosensors that use graphene are particularly attractive as the large surface area, excellent electrical conductivity, and high electron transfer rate [23] can allow highly sensitive biomarker measurements. In such sensors, the binding of biomarker and aptamer molecules induces a change in the carrier concentration of graphene, which is reflected by a measurable device output for the determination of the biomarker concentration.

Aptamer-based graphene affinity nanosensors have been used for biomarker measurements with relevance to clinical diagnostics [24,25], environmental monitoring [26,27], and other related applications [28,29]. Such devices, however, have thus far been mostly limited to operating in conditioned buffers and are not yet applicable to physiological media as required for clinical applications. In physiological media (e.g., serum, urine, sweat, and tears), nonspecific background molecules can adsorb to the graphene surface and render the sensors ineffective. An effective strategy to address this issue is to protect the graphene surface with an anti-adsorption coating to reduce nonspecific binding. For example, physisorption of molecules such as bovine serum albumin (BSA) [30], Tween 20 [31], and Nafion [32] have been used to mitigate nonspecific adsorption to graphene. While simple to implement, physisorption-based molecular coatings can, however, be susceptible to detachment from surfaces, leading to a lack of reliability.

In comparison, protecting the graphene surface with the polymer PEG via a more robust attachment strategy can provide a more reliable reduction in nonspecific binding. PEG achieves suppression of nonspecific adsorption by forming well-solvated brushes as a high activation barrier [33] while also providing the added benefit of reducing charge screening on the graphene surface in media of high ionic strength to improve the sensitivity in biomarker measurements [34]. For proof of principle of using PEG to reduce nonspecific binding in physiological media, we previously reported PEG-coated aptameric graphene nanosensors with which proteins were captured in serum and then measured in conditioned buffer solution [35] or with which proteins were captured and measured within artificial tears [36]. However, there has not yet been any investigation of how aptamers and PEG attached to the graphene surface impact the sensor behavior in physiological media or how these surface functionalization schemes and parameters can be chosen to optimize the performance of aptameric graphene nanosensors.

This paper aims to optimize the surface modification of graphene nanosensors for sensitive and reliable operation in undiluted physiological media where a complex variety of matrix molecules are present in addition to the intended biomarker. We examine how aptamer and PEG attachment schemes and parameters impact the reduction in nonspecific binding and then determine the optimal modification of graphene surfaces to produce aptameric graphene nanosensors that operate effectively in undiluted physiological media. Unlike our previous proof-of-concept work, where an arbitrarily selected, non-optimal PEG coating was used without attempting to understand the impact of the surface modification scheme choices, here, we systematically investigate the effects of the schemes and parameters of surface functionalization on the behavior of aptameric graphene nanosensors. These include the impact of serial or parallel PEG and the aptamer attachment scheme, PEG molecular weight, PEG surface density, and aptamer surface density on the sensor performance, such as the responsivity to biomarker concentration changes, and importantly, for operation in physiological media, the ability to reject nonspecific binding to interfering matrix molecules. We then use the understanding from this parametric study to identify graphene nanosensor designs that are optimally functionalized with PEG and aptamers for highly sensitive and specific biomarker measurements in undiluted physiological media.

The experimental results show that nanosensors functionalized with higher PEG molecular weights and higher PEG and aptamer surface densities through a serial PEG–aptamer modification scheme provide more effective minimization of nonspecific binding and offer enhanced responsivity for biomarker measurements in physiological media. In particular, devices serially modified with 5000 Da PEG at 15 mM and a 94 nt DNA aptamer at 500 nM allowed specific measurement of C-reactive protein (CRP), used as a representative biomarker. CRP is a systemic inflammation marker and is routinely measured for assessment of infection, chronic inflammatory disease monitoring, and cardiovascular risk stratification [37]. Its physiological concentration ranges from 10 s of pM in non-inflammatory conditions to several orders of magnitude higher during acute inflammatory and pathological conditions [38]. An LOD down to 27 pM in undiluted human serum was achieved, representing an improvement by a factor of approximately 70 to 1000 compared to the previously reported CRP measurement results [39,40]. Thus, nanosensors can potentially be used to detect CRP concentrations that are only moderately outside the normal range for identification of early-stage and subtle inflammatory changes.

## 2. Results and Discussion

### 2.1. Experimental Design

**Figure 1:**
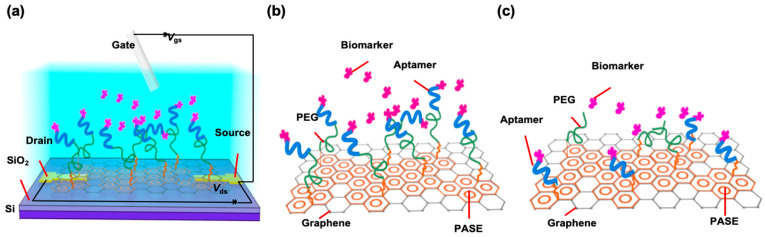
An aptamer-based graphene nanosensor. (**a**) The device is a field-effect transistor with graphene as the conducting channel. (**b**) An aptamer is attached to PEG, which is in turn attached to graphene. (**c**) The aptamer and PEG are both directly attached to graphene.

The nanosensor is an electrolyte-gated field-effect transistor (FET), in which graphene, serving as the conducting channel, is modified with PEG to minimize nonspecific binding and a nucleic acid aptamer to specifically recognize the target biomarker (Figure 1a) [35,36]. The device was fabricated on a Si/SiO_2_ wafer as a substrate. Ti (5 nm) and Au (50 nm) were deposited and patterned to form the source, drain, and gate electrodes. Chemical vapor deposition (CVD) graphene was then transferred onto the electrodes and patterned to form the conducting channel. 1-Pyrenebutanoic acid succinimidyl ester (PASE) was modified on the surface via π-π stacking with graphene [36]. Two schemes were then used to modify the graphene–PASE surface with PEG and the aptamer. In one scheme (serial attachment), the aptamer was attached to PEG, which was in turn attached to the graphene surface (Figure 1b); in the other scheme (parallel attachment), the aptamer and PEG were both directly attached to the graphene surface (Figure 1c). Further details of the design, fabrication, and surface modification of the nanosensor are provided in the Supplementary Information.

The experimental design involved a systematic study of the key surface modification schemes and parameters to understand how they influence the minimization of nonspecific binding for measurements of biomarker analytes in undiluted physiological media. We first compared serial versus parallel attachment of PEG and aptamer molecules to graphene to determine their relative efficacy for PEG to reject nonspecific binding and for the aptamer–analyte binding to be transduced into a measurable conductivity change in the graphene. Second, we examined the effect of the molecular weight of PEG. This parameter determined the thickness of the PEG nanolayer for the protection of graphene from nonspecific binding while also impacting the Debye screening length on the graphene surface. Third, we investigated the effect of the PEG surface density resulting from treating the graphene surface with varying concentrations of PEG. This parameter influenced the formation of a dense, well-solvated PEG nanolayer on the graphene surface, which impacted the effectiveness of nonspecific binding rejection and determined the number of available aptamer recognition sites per unit area. The fourth key parameter studied was the aptamer surface density resulting from modifying the PEG-saturated graphene surface with the aptamer at varying concentrations. This parameter directly influenced the number of analyte molecules that could be captured per unit area of graphene, determining the strength of the graphene’s response to the presence of the analyte.

We finally used insights from the parametric study to identify the optimal functionalization of the graphene nanosensor by the aptamer and PEG to achieve effective minimization of nonspecific binding and sensitive and specific detection and measurement of biomarkers in complex, undiluted physiological media.

### 2.2. Physical Characteristics and Biomarker Specificity

**Figure 2:**
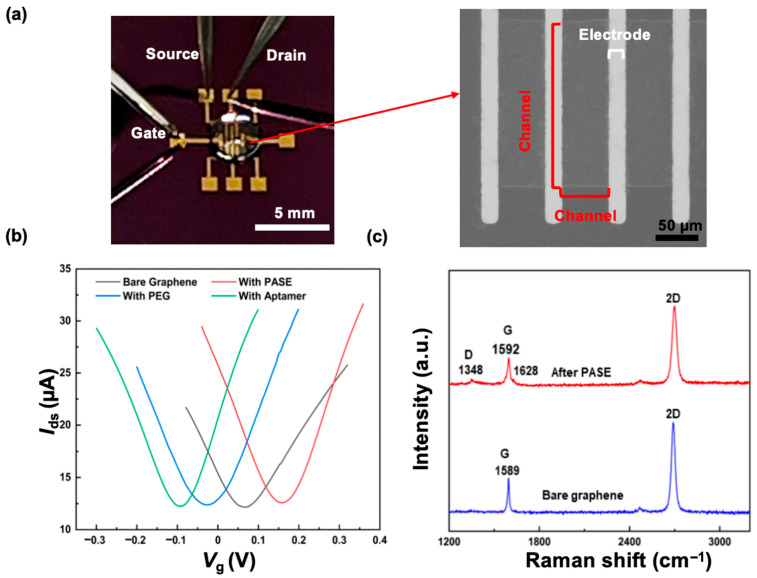
Surface characterization of a nanosensor. (**a**) Photograph of the nanosensor chip in contact with testing probes. Micrograph of the graphene conducting channel of the device. (**b**) Transfer characteristic curve of the chip following sequential surface modification with PASE, PEG, and aptamer. (**c**) Raman spectrum of a graphene sample before and after PASE attachment.

In the experimental setup, drain and source probes of a nanosensor were connected with contact pads, and the drain–source current was recorded by a sourcemeter (Figure 2a). The transfer characteristic curve, i.e., the drain–source current (*I*_ds_) plotted with respect to the gate voltage (*V*_g_), was measured to verify the modification of the device (Figure 2b) [36]. The curve allowed identification of the Dirac point (*V*_Dirac_) as the value of *V*_g_ at which *I*_ds_ reaches the minimum. *V*_Dirac_ shifted from 64.9 to 158.1 mV after PASE was attached to the graphene surface, suggesting that PASE induced p-type doping in the graphene [35,36]. Upon attachment of PEG (e.g., 2000 Da), which induced n-type doping in the graphene [35,36], the Dirac point negatively shifted by −259.86 mV, indicating that PEG was successfully attached to the graphene. The Dirac point shifted to −93 mV after the aptamer attachment, which induced n-type doping in the graphene [35,36], indicating that the aptamer was successfully attached to the graphene.

The Dirac point shifts observed above reflect carrier concentration changes in the graphene due to molecular events on the surface. At the Dirac point (i.e., *V*_g_ = *V*_Dirac_), the electron and hole densities reach an equilibrium in the bulk of the graphene, which hence achieves charge neutrality [41]. When PASE, PEG, or aptamer molecules are present on the surface, the charge carried by these molecules (aptamer) or the interaction of these molecules (PASE and PEG) with the surface induces carrier concentration changes in the graphene, resulting in excess free electrons (n-doping, aptamer and PEG) or holes (p-doping, PASE) [35,36]. Thus, the Dirac point must experience a negative shift to electrostatically deplete the excess electrons (aptamer and PEG) or a positive shift to compensate for the excess electron deficiency (PASE), thereby reaching a new electron–hole equilibrium in the channel.

The graphene surface and its modification were additionally investigated using Raman spectroscopy (Figure 2c). The Raman spectrum revealed the existence of a G band (1589 cm^−1^) and a 2D band (2679 cm^−1^), with an intensity ratio (2D band to G band) of 2.5, confirming the surface to be that of monolayer graphene [42]. After PASE attachment, the split of the G band (1589 and 1628 cm^−1^) and noticeable D band (1348 cm^−1^) appeared in the Raman spectrum, reflecting that PASE induced p-type doping in the graphene. This confirmed the coupling between the graphene and pyrene groups on PASE.

The specificity of the nanosensors to target biomarkers was investigated. C-reactive protein (CRP), a protein indicative of inflammation due to infection, injury, or chronic disease [43], was used as a representative biomarker, while pentraxin3 (PTX 3), a related inflammatory protein [44] and a member of the pentraxin family sharing structural similarity with CRP, was used as a control.

**Figure 3:**
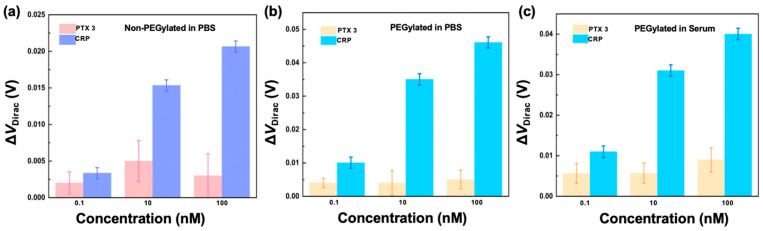
Biomarker specificity. (**a**) The sensing response of non-PEGylated nanosensors to CRP over PTX 3 (control target) in PBS. The sensing response of PEGylated nanosensors to CRP compared to PTX 3 measurement in (**b**) PBS and (**c**) human serum, respectively.

Non-PEGylated nanosensors were first tested (Figure 3a). The devices modified with the CRP aptamer (500 nM) were immersed in a solution of CRP and PTX 3 for measurements of the shift in the Dirac point, Δ*V*_Dirac_ = *V*_Dirac_ − *V*_Dirac,0_, where *V*_Dirac,0_ is the Dirac point of the device at CRP concentrations of 0. The response of the device to PTX 3 was 1.3, 4.8, and 2.5 mV as the PTX 3 concentration was at 0.1, 10, and 100 nM, which was only 41%, 31%, and 12% of that of the device to CRP at the corresponding concentrations. This indicated that the aptamer-functionalized nanosensor modified with the CRP aptamer was specific to CRP, the target biomarker.

The PEGylated nanosensors with serial and parallel surface modification schemes were then used for CRP and PTX 3 measurements in phosphate-buffered saline (PBS) and undiluted human serum, respectively (Figure 3b,c). For a device with the serial modification scheme, the Dirac point shift was measured to be 10, 35, and 48 mV when the concentration of CRP was at 0.1, 10, and 100 nM. In comparison, the response of the device with a serial surface modification scheme to PTX 3 at these concentrations was 4.1, 4.0, and 5.2 mV, which was 40%, 11%, and 11% of that of the device to CRP at the corresponding concentrations. A PEGylated device with a serial modification scheme was next tested in undiluted human serum. The Dirac point shift was measured to be 11, 21, and 40 mV when the concentration of CRP was at 0.1, 10, and 100 nM, respectively. In comparison, the response of the device to PTX 3 at these concentrations was 5.6, 5.7, and 9.0 mV, or 51%, 27%, and 23% of the Dirac point shift for the device to CRP at these concentrations. Thus, the response of the CRP aptamer-functionalized nanosensor to PTX 3 was considerably smaller than that to CRP in both PBS and human serum, suggesting that the device was specific to CRP.

The specificity of the nanosensors with PEG and aptamer modification in parallel was next tested against PTX 3 and CRP in PBS and undiluted human serum (Figure S1a,b). As the concentration of PTX 3 was changed from 0.1 to 100 nM in PBS, the Dirac point shift of the device varied from 1.6 to 5.1 mV, or from 27% to 15% of the device response to CRP in the same concentration range. In addition, the response of the device to PTX 3 in human serum ranged from 1.1 to 5.0 mV as the PTX 3 concentration varied from 0.1 to 100 nM. These were 28% to 19% of the device’s Dirac point shift in response to CRP at these concentrations. Thus, with a response to PTX 3 much smaller than that to CRP, the nanosensor with parallel PEG and aptamer modification was considered specific to CRP.

### 2.3. Effects of Graphene Surface Modification Schemes and Parameters

We investigated surface modification schemes and parameters that impact the reduction in nonspecific binding in PBS and undiluted physiological media, and the key optimization parameters are summarized in Table 1.

#### 2.3.1. Behavior of Non-PEGylated Nanosensors in PBS and Human Serum

We first investigated the responses to CRP, respectively, in PBS and undiluted human serum, of nanosensors that were modified with the aptamer at 500 nM but not protected with PEG.

**Figure 4:**
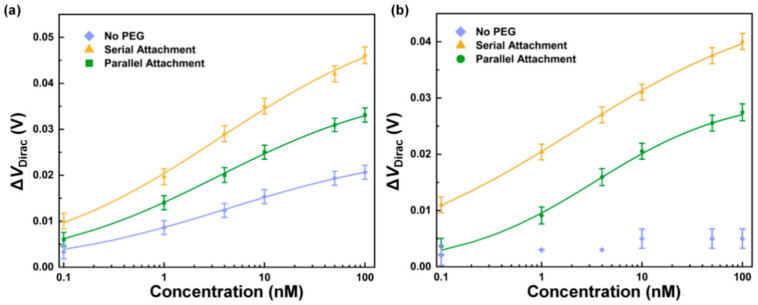
Effects of the surface modification scheme. The Dirac point shift as a function of CRP concentration for devices with parallel and serial modification schemes in (**a**) PBS and (**b**) human serum, respectively.

The transfer characteristic curve was obtained with a non-PEGylated nanosensor first exposed to CRP in PBS. As the concentration of CRP increased from 0.1 to 100 nM, the Dirac point decreased from −83 to −104 mV (Figure 4a). This can be explained by the nanosensor’s transduction mechanism: the guanine-rich aptamer binds with the biomarker and folds into a stable and compact G-quadruplex formation [45]. As a result, the complex of a negatively charged aptamer with the protein was brought closer to the graphene surface [46,47], thus redistributing the carrier concentration in graphene and resulting in the measured decrease in the Dirac point with the concentration of CRP.

We then tested a non-PEGylated nanosensor in undiluted human serum. When the concentration of CRP ranged from 0.1 to 100 nM, the Dirac point shift of the non-PEGylated device experienced a change of 5.3 mV compared to 21 mV of the device in PBS. Thus, the response of the non-PEGylated device to CRP in undiluted human serum was considered insignificant, which could be attributed to nonspecific binding in human serum.

#### 2.3.2. Effects of the Graphene Surface Modification Scheme

The impact of different surface modification schemes on the behavior of nanosensors in PBS and undiluted human serum was investigated. The nanosensors were modified through serial and parallel surface modification schemes with PEG of molecular weight 2000 Da at a concentration of 15 mM and with the aptamer at a concentration of 500 nM.

The nanosensors with serial and parallel surface modification schemes were first tested in PBS buffer (Figure 4a). The transfer characteristic curves were measured (Figure S2a,b). As the concentration of CRP increased from 0.1 to 100 nM, the Dirac point shift of the device with a serial surface modification scheme changed by 38 mV, which was 1.4 times that of the device with a parallel surface modification scheme (27 mV). The higher signal levels from serial PEG and the aptamer attachment could be attributed to the beneficial role of PEG in enhancing the performance of graphene FET sensors [48]. In the serial attachment, PEG has access to a larger number of reactive sites on PASE in the absence of competition from aptamers to form a dense, continuous isolation layer. In comparison, in the parallel attachment, introducing PEG and aptamers simultaneously leads to competition for reactive sites on PASE, leading to reduced surface coverage by PEG.

These results were further compared with the non-PEGylated nanosensor results above. The changes in the Dirac point shifts of the non-PEGylated devices were 78% and 55% of those of the PEGylated devices with parallel and serial surface modification schemes, respectively, when the concentration of CRP changed from 0.1 to 100 nM. The PEGylated nanosensors outperformed the non-PEGylated nanosensor in the CRP measurement, regardless of surface modification scheme, reflecting that PEG can prevent nonspecific binding and increase the Debye length to enhance the responsivity of the PEGylated nanosensor for the CRP measurement.

Nanosensors with the different surface modification schemes were then tested in undiluted human serum (Figure 4b). Transfer characteristic curves of the nanosensors were measured (Figure S2c,d). When the CRP concentration increased from 0.1 to 100 nM, the Dirac point shift of the device with a serial surface modification scheme exhibited a change of 29 mV, which was 25% more than that of the device with a parallel surface modification scheme (23 mV). Thus, for measurements in human serum, the nanosensor with the serial modification strategy also exhibited a higher sensitivity compared to the device with the parallel modification strategy. In comparison with the non-PEGylated nanosensor, the PEGylated nanosensors with either serial surface modification schemes or parallel surface modification schemes succeeded in measuring CRP in undiluted human serum, indicating effective suppression of nonspecific adsorption of matrix molecules.

Starting with the next section, we will focus on the scheme of serial aptamer–PEG attachment to graphene and study the effects of the key surface modification parameters, including PEG molecular weight, PEG surface density, and aptamer surface density, on the nanosensor behavior in undiluted human serum. Incubating sensor chips with PEG of 2000 Da molecular weight at 15 mM concentration and with the aptamer at 500 nM concentration will be used as a set of standard parameter values. One parameter at a time will be varied from its standard value in each parametric study.

#### 2.3.3. Effects of PEG Molecular Weight

The effects of PEG molecular weight on the characteristics of nanosensors in PBS and undiluted human serum were investigated. With reference to the standard surface modification parameters (2000 Da PEG at 15 mM and an aptamer at 500 nM), the graphene surfaces were modified with PEG of additional molecular weights of 1000 and 5000 Da. The other modification parameters remained unchanged.

**Figure 5:**
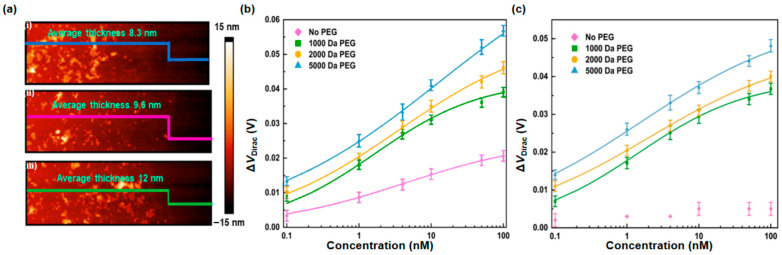
Effects of PEG molecular weight. (**a**) AFM images of the graphene surfaces modified with PEG of molecular weight at (**i**) 1000, (**ii**) 2000, and (**iii**) 5000 Da, respectively. The Dirac point shift as a function of CRP concentration for the devices modified with PEG of different molecular weights in (**b**) PBS and (**c**) human serum, respectively.

The graphene surfaces modified with PEG at different molecular weights were examined using atomic force microscopy (AFM) (Figure 5a). The thickness of the PASE-attached graphene layer was 4 nm. After PEG attachment, the thickness of the graphene layers modified with PASE followed by PEG was 8.3, 9.6, and 12 nm when the molecular weight of PEG was 1000, 2000, and 5000 Da, respectively. The thickness of the PEG layer increased with the molecular weight of PEG, thereby enabling substantial surface coverage to prevent nonspecific binding [49] and extending the Debye screening length.

The nanosensors, modified with PEG at the varying molecular weights, were then tested for the CRP measurement in PBS (Figure 5b). The transfer characteristic curves were measured (Figure S3a,b). As the concentration of CRP increased from 0.1 to 100 nM, the Dirac point shift of the devices modified with PEG of molecular weight at 1000 and 5000 Da changed by 32 and 43 mV, respectively. These results were, respectively, 84% and 113% of those of the device modified with 2000 Da PEG, respectively. These results were attributed to the high molecular weight of PEG, which can prevent nonspecific binding [50] and increase the Debye screening length, thereby improving the device’s responsivity for the CRP measurement.

The PEGylated nanosensor outperformed the non-PEGylated nanosensor in responding to CRP in PBS. In a CRP concentration range of 0.1 to 100 nM, the changes of the Dirac point shifts for the non-PEGylated devices were 66%, 55%, and 49% of those for the PEGylated devices modified with PEG of molecular weight at 1000, 2000, and 5000 Da, respectively, which were attributed to the ability of PEG to suppress nonspecific binding and increase the Debye screening length.

The nanosensors modified with PEG at different molecular weights were next tested in undiluted human serum (Figure 5c), and the transfer characteristic curves were measured (Figure S3c,d). As the CRP concentration was changed from 0.1 to 100 nM, the Dirac point shift of the devices modified with PEG of 1000 and 5000 Da experienced a change of 27 and 34 mV, respectively. These were, respectively, 93% and 117% of the shift of the device modified with PEG of 2000 Da. This confirmed that the PEGylated device can detect CRP in undiluted human serum, without being limited by the molecular weight of PEG. This result also demonstrated that the device modified with PEG at high molecular weights can effectively prevent nonspecific binding and increase the Debye screening length in undiluted human serum compared to low molecular weights, thereby achieving the CRP measurement with a high responsivity. In comparison with the non-PEGylated device, the PEGylated devices successfully detected CRP in undiluted human serum, regardless of the molecular weight of PEG, reflecting the ability of PEG to prevent nonspecific binding and increase the Debye screening length in undiluted human serum.

#### 2.3.4. Effects of the PEG Surface Density

We then investigated the effects of PEG surface density on the behavior of nanosensors in PBS and undiluted human serum, respectively. With reference to the standard surface modification parameters (2000 Da PEG at 15 mM and aptamer at 500 nM), the graphene surfaces were additionally modified with 2000 Da PEG at 1 and 5 mM. The other modification parameters remained unchanged.

**Figure 6:**
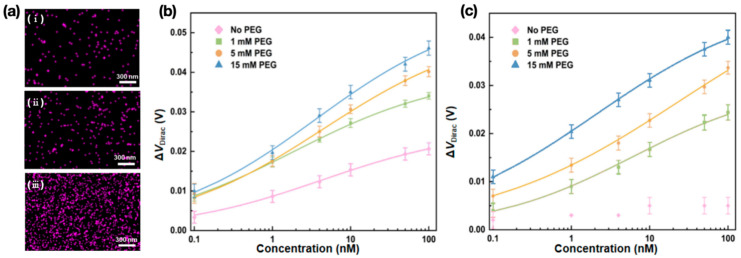
Effects of PEG density on the graphene surfaces. (**a**) EDS characterization of the graphene surfaces modified with PEG at the concentrations of (**i**) 1, (**ii**) 5, and (**iii**) 15 mM, respectively. Pink dots represent the distribution of phosphorus on the graphene surfaces. The Dirac point shift of nanosensors with different PEG surface densities in response to CRP in (**b**) PBS and (**c**) human serum, respectively.

To control the density of PEG immobilized on the graphene surfaces, the surfaces were treated with PEG solutions of different concentrations. The surfaces were then immersed in aptamer solution with a constant concentration (500 nM), attaining different aptamer surface densities based on different PEG surface densities. Energy-dispersive X-ray spectroscopy (EDS) was employed to examine the aptamer density on the graphene surfaces, which can reflect the corresponding PEG surface density (Figure 6a). Phosphorus was observed on the graphene surfaces with different PEG densities, as reflected by pink-colored dots in the elemental maps given in the form of EDS images. As phosphorus is contained only in the aptamer but not in other surface-attached components, the phosphorus-representing, pink-colored dots in the EDS images allowed for visualization of the density of aptamer–PEG complexes immobilized on the graphene surfaces. Therefore, in the EDS elemental maps, the PEG surface density can be characterized by the phosphorus–coverage ratio, i.e., the ratio of the area covered with pink dots to the overall area of the graphene surface [24]. The ratio was calculated to be 2.5%, 7.5%, and 15%, respectively, as the concentration of PEG attached to the graphene surface was 1, 5, and 15 mM, respectively. The PEG density on the graphene surface increased with the concentration of PEG attached to the surface, which provided more available aptamer recognition sites, thereby increasing the possibility of binding between the aptamer and biomarker.

The PEGylated nanosensors with different PEG surface densities were then tested for CRP in PBS (Figure 6b), and the transfer characteristic curves were measured (Figure S4a,b). As the concentration of CRP increased from 0.1 to 100 nM, the Dirac point shift of the devices modified with PEG at the concentrations of 1 and 5 mM changed by 30 and 32 mV, respectively. These were, respectively, 79% and 84% of that of the device modified with PEG at the concentration of 15 mM PEG, reflecting that the denser, well-solvated brushes formed by PEG with high density can effectively prevent nonspecific binding. The nanosensor modified with a higher PEG at a concentration of 20 mM was further used for the CRP measurement (Figure S4c). In a CRP concentration range of 0.1 to 100 nM, the Dirac point shift exhibited a change of 38 mV, which agreed with that of the device modified with PEG at a concentration of 15 mM, suggesting saturation of the surface modified with PEG at a concentration of 15 mM.

We then compared the behavior of the PEGylated nanosensor with the non-PEGylated nanosensor. The PEGylated device outperformed the non-PEGylated device at any given CRP concentration. As the concentration of CRP increased from 0.1 to 100 nM, the changes in the Dirac point shifts of the non-PEGylated devices were 70%, 66%, and 55% of those of the devices modified with PEG at the concentrations of 1, 5, and 15 mM, respectively. This was attributed to the well-solvated brushes formed by PEG molecules, resulting in a high activation barrier on the graphene surface to prevent nonspecific binding.

The nanosensors with different PEG surface densities were then used to detect CRP in undiluted human serum (Figure 6c), and the corresponding transfer characteristic curves were measured (Figure S4d,e). As the CRP concentration changed from 0.1 to 100 nM, the Dirac point shift of the devices modified with PEG at the concentrations of 1 and 5 mM changed by 21 and 27 mV, which were, respectively, 72% and 93% of that of the device modified with PEG at the concentration of 15 mM. These results illustrated that devices with a high PEG surface density responded more strongly to CRP in undiluted human serum. In comparison with the non-PEGylated device, the PEGylated device, regardless of the PEG surface density, succeeded in the CRP detection in undiluted human serum, reflecting the capability of PEG to suppress nonspecific binding and expand the Debye length.

#### 2.3.5. Effects of the Aptamer Surface Density

The effects of the aptamer surface density on nanosensor behavior in PBS and undiluted human serum, respectively, were finally investigated. With reference to the standard surface modification parameters (2000 Da PEG at 15 mM and aptamer at 500 nM), the graphene surfaces were additionally modified with the aptamer at concentrations of 50 and 150 nM. The other modification parameters remained unchanged.

**Figure 7:**
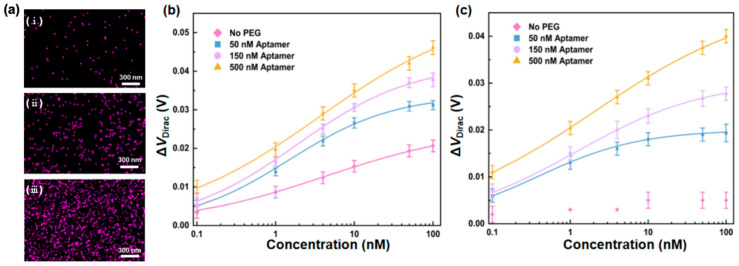
Effects of aptamer density on the graphene surfaces. (**a**) EDS elemental maps are shown of the graphene surfaces modified with CRP aptamer at the concentrations of (**i**) 50, (**ii**) 150, and (**iii**) 500 nM, respectively. Pink dots represent the distribution of phosphorus on the graphene surfaces. Dirac point shifts of the nanosensors with different aptamer surface densities for CRP measurements in (**b**) PBS and (**c**) human serum, respectively.

To control the density of aptamer molecules immobilized on the graphene surfaces, the surfaces were treated with aptamer solutions of different concentrations, and EDS was employed to examine the aptamer density on the graphene surfaces (Figure 7a). The ratio of the area covered with pink dots to the overall area of the graphene was calculated to be 1.9%, 6.5%, and 15% when the graphene surfaces were modified with the aptamer at 50, 150, and 500 nM, respectively. The aptamer surface density increased with the concentration of the aptamer molecule, thereby providing more available CRP recognition sites for CRP recognition.

The PEGylated nanosensors functionalized with different concentrations of the aptamer were then used for the CRP measurement in PBS (Figure 7b), and the transfer characteristic curves were measured (Figure S5a,b). When the surfaces were coated with PEG and functionalized with the aptamer molecule at varying densities, the behavior of the devices was different. As the CRP concentration increased from 0.1 to 100 nM, the Dirac point shift of the devices modified with the aptamer at the concentrations of 50 and 150 nM experienced a change of 28 and 31 mV, respectively. These were, respectively, 74% and 82% of the shift of the device modified with the aptamer at a concentration of 500 nM. That is, a stronger response of the nanosensor was observed at a higher aptamer surface density, which was attributed to the increased availability of CRP recognition sites for CRP on the graphene surface. The nanosensor modified with the aptamer at a higher concentration of 1000 nM was further used for the CRP measurement (Figure S5c). When the concentration of CRP changed from 0.1 to 100 nM, the Dirac point shift experienced a change of 38 mV, comparable to that of the aptamer at the concentration of 500 nM, suggesting saturation of the aptamer at 500 nM.

Next, we compared the behavior of the PEGylated nanosensor with the non-PEGylated nanosensor. In a CRP concentration range of 0.1 to 100 nM, the change in the Dirac point shift of the PEGylated devices modified with the aptamer at the concentrations of 50, 150, and 500 nM was 1.3, 1.5, and 1.8 times that of the non-PEGylated device, which could be attributed to the previously reported finding that PEG modification may effectively increase the Debye screening length on the graphene surface.

The nanosensors with different aptamer surface densities were next tested in undiluted human serum (Figure 7c). The transfer characteristic curves were measured (Figure S5d,e). When the concentration of CRP ranged from 0.1 to 100 nM, the Dirac point shift of the devices modified with the aptamer at the concentrations of 50 and 150 nM exhibited a change of 14 and 21 mV, respectively, which were 48% and 72% of that of the device modified with the aptamer at the concentration of 500 nM. This confirmed that the nanosensor with high aptamer surface density can achieve a stronger output for the CRP measurement in undiluted human serum. In comparison with the non-PEGylated nanosensor, CRP can be successfully measured with the PEGylated nanosensors, reflecting that PEG can suppress nonspecific adsorption in undiluted human serum.

### 2.4. Discussion: Optimal Surface Functionalization for the Nanosensor

The results above indicated that surface modification with PEG rejected nonspecific binding in physiological media. This was attributed to the formation of a protective layer on the graphene surface by PEG, which reduced nonspecific adsorption of nontarget molecules to the surface [51]. The extent to which nonspecific binding was reduced depended on surface modification scheme [52], PEG molecular weight, PEG surface density, and aptamer surface density, which were key parameters for the surface modification. Nanosensors with a serial surface modification scheme outperformed nanosensors with a parallel surface modification scheme. Compared to the parallel surface modification scheme, the serial surface modification scheme can provide more exposure of aptamer recognition sites and cover more sites on the graphene surface to prevent nonspecific binding [35,53]. More possibilities for CRP to bind with the aptamer molecule and less nonspecific binding induced by background molecules allowed a stronger measurable signal for the CRP measurement. Indeed, as the concentration of CRP ranged from 0.1 to 100 nM, the Dirac point shift of the devices with a parallel surface modification scheme changed by 27 mV in PBS and 23 mV in human serum, respectively (Figure 4a,b), or 71% and 79% of the shifts of the devices with a serial surface modification scheme in PBS and human serum.

Nanosensors modified with PEG of a higher molecular weight outperformed the nanosensors modified with PEG of a lower molecular weight. PEG with a higher molecular weight can more effectively protect the graphene surface for enhanced inhibition of nonspecific binding. As the CRP concentration changed from 0.1 to 100 nM, the Dirac point shift of the devices modified with PEG at the molecule weight of 5000 Da exhibited a change of 43 and 34 mV in PBS and human serum, which were 1.3 and 1.1 times those of the devices modified with PEG at the molecular weight of 1000 Da PEG (Figure 5b,c).

As nanosensors with a higher PEG surface density yielded a larger output, this investigation allowed us to identify an ideal PEG concentration for surface modification, beyond which the increase in the PEG surface density would be diminished. Higher PEG surface densities created a denser hydrated layer that helped to reduce nonspecific binding and improve the availability of aptamer recognition sites, and sequentially, CRP was attached. The devices modified with PEG at the concentration of 15 mM outperformed the devices modified with PEG at concentrations of 1 and 5 mM for the CRP measurements in both PBS and human serum (Figure 6b,c). For example, at a CRP concentration of 10 nM, the Dirac point shifts of the devices modified with PEG at the concentration of 15 mM were 1.2 and 1.4 times those for the devices modified with PEG at the concentration of 1 mM for the CRP measurements in PBS and human serum, respectively.

As higher aptamer surface densities would generally result in higher responses, this investigation allowed us to identify an ideal aptamer concentration for surface modification, beyond which the increase in the aptamer surface density would be diminished. The high aptamer surface density can provide a larger number of CRP recognition sites, increasing the probability of capturing the biomarker. As the CRP concentration increased from 0.1 to 100 nM, the Dirac point shift of the devices modified with the aptamer at the concentration of 50 nM exhibited a change of 28 and 14 mV in PBS and human serum, respectively, which were 74% and 48% of that of the devices modified with the aptamer at the concentration of 500 nM (Figure 7b,c).

In summary, nanosensors serially functionalized with PEG at a high molecular weight (5000 Da in our experiments) and a high surface density (15 mM) and with the aptamer at a high surface density (500 nM) provided highly effective minimization of nonspecific binding and strongly enhanced responsivity for biomarker measurements in human serum. These devices allowed sensitive and repeatable measurements of CRP in undiluted human serum with an LOD down to 27 pM. This LOD was a factor of ~70 to 1000 lower than the CRP measurement LODs of the previously reported sensors that used electrochemical [54], guided-mode optical resonance [55], fluorescence [56], and SPR [57] methods. While these results were obtained for CRP, it is expected that the surface modification scheme, PEG molecular weight, PEG surface density, and aptamer surface density can be similarly optimized to enable aptameric graphene nanosensors to measure other biomarkers in human serum and other physiological fluids.

## 3. Conclusions

In this study, we have investigated the impact of aptamer molecules and PEG attachment parameters on reducing nonspecific binding in the pursuit of optimizing graphene surface modifications for sensitive and specific biomarker detection in undiluted physiological media. The attachment parameters investigated include the modification scheme, PEG molecular weight, PEG surface density, and aptamer surface density. The nanosensor itself was configured as a graphene-based field-effect transistor with the graphene surface subject to modification with PEG and aptamers. Insights from our parametric study were used to optimize the modification of the graphene nanosensor to enable sensitive and specific biomarker measurements in undiluted physiological media.

Firstly, we systematically studied the impact of aptamer and PEG attachment methods and parameters on nonspecific binding suppression. Devices with and without PEG protection were tested for their biomarker response in both PBS and undiluted human serum alongside devices with varying surface modification schemes. The PEGylated devices with a serial surface modification scheme outperformed those with a parallel surface modification scheme as well as non-PEGylated devices for biomarker measurements in PBS and undiluted human serum, suggesting enhanced biomarker binding and reduced nonspecific interactions due to PEGylation.

We then investigated the impact of PEG molecule weight on the behavior of the nanosensors. AFM analysis of PEG-modified surfaces with different molecular weights revealed that graphene layer thickness increased from 4 to 8.3, 9.6, and 12 nm for PEG of 1000, 2000, and 5000 Da, respectively, extending Debye screening length to reduce nonspecific binding. The devices modified with PEG at high molecular weight (5000 Da) showed greater Dirac point shifts in the biomarker measurement in both PBS and undiluted human serum when compared to lower molecular weights and to non-PEGylated devices. These results demonstrate the capability of PEG in preventing nonspecific binding and extending Debye screening length.

The impact of PEG surface density on the behavior of nanosensors was next investigated. EDS analysis of aptamer surface density, mirroring PEG density, showed a maximum phosphorus coverage of 15% on the graphene surface modified with PEG at a high concentration (15 mM), increasing aptamer recognition sites for biomarker interactions. The PEGylated devices with the high PEG surface density yielded large Dirac point shifts in PBS and undiluted human serum, reflecting denser PEG brushes effectively preventing nonspecific binding while outperforming the non-PEGylated devices.

Finally, the impact of aptamer surface density on the behavior of the nanosensors was investigated. EDS analysis revealed that the phosphorus–coverage ratio (representing aptamer surface densities) increased with the concentration of the aptamer (1.9%, 6.5%, and 15% for 50, 150, and 500 nM aptamer concentrations) attached on the graphene surface, suggesting that higher concentrations of attached aptamers provide an increased number of biomarker recognition sites. PEG protection, along with high aptamer surface density, resulted in the best observed performance, consistently outperforming the devices with no PEG protection. This could be attributed to enhanced biomarker recognition sites and increased Debye screening length on the graphene surface by PEG modification.

The studies above have shown that nanosensors modified with PEG of suitably high molecular weight (e.g., 5000 Da) at a sufficiently high surface density (e.g., 15 mM), and with the aptamer at a sufficiently high surface density (e.g., 500 nM) through a serial modification scheme, can effectively suppress nonspecific binding and enhance the responsivity of the biomarker measurement. The measurement of CRP in undiluted human serum achieved a limit of detection as low as 27 pM, which was a factor of ~70 to 1000 lower than the previously reported LODs for CRP.

These results demonstrate that the optimized PEGylated nanosensor offers enhanced sensitivity and specificity and can be potentially used for the measurement and monitoring of disease biomarkers in complex physiological solutions through optimizing sensor design.

## Figures and Tables

**Table 1:** Parameters used in graphene surface functionalization.

Parameters	Value
Attachment scheme	Serial, Parallel
PEG molecular weight	1000, 2000, 5000 Da
PEG concentration	1, 5, 15 mM
Aptamer concentration	50, 150, 500 nM

## Data Availability

The original contributions presented in this study are included in the article/Supplementary Material. Further inquiries can be directed to the corresponding author.

## Supplementary Materials

The following supporting information can be downloaded at https://www.mdpi.com/article/10.3390/s26020744/s1, Figure S1. The sensing response of the PEGylated nanosensor with parallel modification scheme to CRP and control protein (PTX 3) in (a) PBS and (b) human serum, respectively. Figure S2. Responses of the nanosensors with different surface modification schemes. Transfer characteristic curves in response to CRP for the PEGylated nanosensors with (a) serial and (b) parallel surface modification schemes in PBS. Transfer characteristics curves in response to CRP for the PEGylated nanosensors with (c) serial and (d) parallel surface modification schemes in human serum. Figure S3. Responses of the nanosensors modified with PEG of different molecule weights. Transfer characteristic curves in response to CRP for the PEGylated nanosensors modified with PEG of molecule weights (a) 1000 and (b) 5000 Da in PBS. Transfer characteristics curves in response to CRP for the PEGylated devices modified with PEG of molecule weights (c) 1000 and (d) 5000 Da in undiluted human serum. Figure S4. Responses of the nanosensors with different PEG surface densities. Transfer characteristic curves in response to CRP for the PEGylated devices modified with PEG of concentrations at (a) 1, (b) 5 and (c) 20 mM in PBS. Transfer characteristics curves in response to CRP for the PEGylated devices modified with PEG of concentrations at (d) 1 and (e) 5 mM in undiluted human serum. Figure S5. Responses of the nanosensors with different aptamer surface densities. Transfer characteristic curves in response to CRP for the PEGylated nanosensors modified with the aptamer at the concentrations of (a) 50, (b) 150 and (c) 1000 nM in PBS. Transfer characteristics curves in response to CRP for the PEGylated devices modified with the aptamer at the concentrations of (d) 50 and (e) 150 nM in undiluted human serum.

## References

[1] Yakoh A., Pimpitak U., Rengpipat S., Hirankarn N., Chailapakul O., Chaiyo S. (2021). Paper-based electrochemical biosensor for diagnosing COVID-19: Detection of SARS-CoV-2 antibodies and antigen. Biosens. Bioelectron..

[2] Elledge S.K., Zhou X.X., Byrnes J.R., Martinko A.J., Lui I., Pance K., Lim S.A., Glasgow J.E., Glasgow A.A., Turcios K. (2021). Engineering luminescent biosensors for point-of-care SARS-CoV-2 antibody detection. Nat. Biotechnol..

[3] Hwang H.J., Ryu M.Y., Park C.Y., Ahn J., Park H.G., Choi C., Ha S.-D., Park T.J., Park J.P. (2017). High sensitive and selective electrochemical biosensor: Label-free detection of human norovirus using affinity peptide as molecular binder. Biosens. Bioelectron..

[4] Wang M., Li L., Zhang L., Zhao J., Jiang Z., Wang W. (2022). Peptide-Derived Biosensors and Their Applications in Tumor Immunology-Related Detection. Anal. Chem..

[5] Mou Q., Xue X., Ma Y., Banik M., Garcia V., Guo W., Wang J., Song T., Chen L.-Q., Lu Y. (2022). Efficient delivery of a DNA aptamer-based biosensor into plant cells for glucose sensing through thiol-mediated uptake. Sci. Adv..

[6] Wang Z., Hao Z., Yu S., De Moraes C.G., Suh L.H., Zhao X., Lin Q. (2019). An Ultraflexible and Stretchable Aptameric Graphene Nanosensor for Biomarker Detection and Monitoring. Adv. Funct. Mater..

[7] Smith R., Duan W., Quarterman J., Morris A., Collie C., Black M., Toor F., Salem A.K. (2019). Surface Modifying Doped Silicon Nanowire Based Solar Cells for Applications in Biosensing. Adv. Mater. Technol..

[8] Presnova G., Presnov D., Krupenin V., Grigorenko V., Trifonov A., Andreeva I., Ignatenko O., Egorov A., Rubtsova M. (2017). Biosensor based on a silicon nanowire field-effect transistor functionalized by gold nanoparticles for the highly sensitive determination of prostate specific antigen. Biosens. Bioelectron..

[9] Alim S., Vejayan J., Yusoff M.M., Kafi A.K.M. (2018). Recent uses of carbon nanotubes & gold nanoparticles in electrochemistry with application in biosensing: A review. Biosens. Bioelectron..

[10] Miao S.S., Wu M.S., Ma L.Y., He X.J., Yang H. (2016). Electrochemiluminescence biosensor for determination of organophosphorous pesticides based on bimetallic Pt-Au/multi-walled carbon nanotubes modified electrode. Talanta.

[11] Alwarappan S., Nesakumar N., Sun D., Hu T.Y., Li C.-Z. (2022). 2D metal carbides and nitrides (MXenes) for sensors and biosensors. Biosens. Bioelectron..

[12] Song M., Pang S., Guo F., Wong M., Hao J. (2020). Fluoride-Free 2D Niobium Carbide MXenes as Stable and Biocompatible Nanoplatforms for Electrochemical Biosensors with Ultrahigh Sensitivity. Adv. Sci..

[13] Tajik S., Dourandish Z., Nejad F.G., Beitollahi H., Jahani P.M., Di Bartolomeo A. (2022). Transition metal dichalcogenides: Synthesis and use in the development of electrochemical sensors and biosensors. Biosens. Bioelectron..

[14] Barua S., Dutta H.S., Gogoi S., Devi R., Khan R. (2018). Nanostructured MoS_2_-Based Advanced Biosensors: A Review. ACS Appl. Nano Mater..

[15] Xu S., Zhan J., Man B., Jiang S., Yue W., Gao S., Guo C., Liu H., Li Z., Wang J. (2017). Real-time reliable determination of binding kinetics of DNA hybridization using a multi-channel graphene biosensor. Nat. Commun..

[16] Justino C.I.L., Gomes A.R., Freitas A.C., Duarte A.C., Rocha-Santos T.A.P. (2017). Graphene based sensors and biosensors. Trends Anal. Chem..

[17] Zhu L., Miao M., Shao X., Du Z., Huang K., Luo Y., Xu W. (2019). A Universal Electrochemical Biosensor Using Nick-HCR Nanostructure as Molecular Gate of Nanochannel for Detecting Chromium(III) Ions and MicroRNA. Anal. Chem..

[18] Zhou Y., Tang L., Zeng G., Zhang C., Zhang Y., Xie X. (2016). Current progress in biosensors for heavy metal ions based on DNAzymes/DNA molecules functionalized nanostructures: A review. Sens. Actuators B Chem..

[19] Liang M., Li Z., Wang W., Liu J., Liu L., Zhu G., Karthik L., Wang M., Wang K.-F., Wang Z. (2019). A CRISPR-Cas12a-derived biosensing platform for the highly sensitive detection of diverse small molecules. Nat. Commun..

[20] Thompson I.A.P., Saunders J., Zheng L., Hariri A.A., Maganzini N., Cartwright A.P., Pan J., Yee S., Dory C., Eisenstein M. (2023). An antibody-based molecular switch for continuous small-molecule biosensing. Sci. Adv..

[21] Ray S., Panjikar S., Anand R. (2017). Structure Guided Design of Protein Biosensors for Phenolic Pollutants. ACS Sens..

[22] Zhao Y., Chen J., Hu Z., Chen Y., Tao Y., Wang L., Li L., Wang P., Li H.-Y., Zhang J. (2022). All-solid-state SARS-CoV-2 protein biosensor employing colloidal quantum dots-modified electrode. Biosens. Bioelectron..

[23] Béraud A., Sauvage M., Bazán C.M., Tie M., Bencherif A., Bouilly D. (2021). Graphene field-effect transistors as bioanalytical sensors: Design, operation and performance. Analyst.

[24] Hao Z., Pan Y., Shao W., Lin Q., Zhao X. (2019). Graphene-based fully integrated portable nanosensing system for on-line detection of cytokine biomarkers in saliva. Biosens. Bioelectron..

[25] Wu D., Yu Y., Jin D., Xiao M.-M., Zhang Z.-Y., Zhang G.-J. (2020). Dual-Aptamer Modified Graphene Field-Effect Transistor Nanosensor for Label-Free and Specific Detection of Hepatocellular Carcinoma-Derived Microvesicles. Anal. Chem..

[26] Li Y., Zhu Y., Wang C., He M., Lin Q. (2019). Selective detection of water pollutants using a differential aptamer-based graphene biosensor. Biosens. Bioelectron..

[27] Farzin L., Shamsipur M., Sheibani S. (2017). A review: Aptamer-based analytical strategies using the nanomaterials for environmental and human monitoring of toxic heavy metals. Talanta.

[28] Verdian A. (2018). Apta-nanosensors for detection and quantitative determination of acetamiprid—A pesticide residue in food and environment. Talanta.

[29] Vishnubhotla R., Ping J., Gao Z., Lee A., Saouaf O., Vrudhula A., Johnson A.T.C. (2017). Scalable graphene aptasensors for drug quantification. AIP Adv..

[30] Wee K.W., Kang G.Y., Park J., Kang J.Y., Yoon D.S., Park J.H., Kim T.S. (2005). Novel electrical detection of label-free disease marker proteins using piezoresistive self-sensing micro-cantilevers. Biosens. Bioelectron..

[31] Chang H.-K., Ishikawa F.N., Zhang R., Datar R., Cote R.J., Thompson M.E., Zhou C. (2011). Rapid, Label-Free, Electrical Whole Blood Bioassay Based on Nanobiosensor Systems. ACS Nano.

[32] Wang Z., Hao Z., Wang X., Huang C., Lin Q., Zhao X., Pan Y. (2021). A flexible and regenerative aptameric graphene–nafion biosensor for cytokine storm biomarker monitoring in undiluted biofluids toward wearable applications. Adv. Funct. Mater..

[33] Kastantin M., Ananthanarayanan B., Karmali P., Ruoslahti E., Tirrell M. (2009). Effect of the Lipid Chain Melting Transition on the Stability of DSPE-PEG(2000) Micelles. Langmuir.

[34] Gao N., Gao T., Yang X., Dai X., Zhou W., Zhang A., Lieber C.M. (2016). Specific detection of biomolecules in physiological solutions using graphene transistor biosensors. Proc. Natl. Acad. Sci. USA.

[35] Wang X., Zhu Y., Olsen T.R., Sun N., Zhang W., Pei R., Lin Q. (2018). A graphene aptasensor for biomarker detection in human serum. Electrochim. Acta.

[36] Wang Z., Dai W., Yu S., Hao Z., Pei R., De Moraes C.G., Suh L.H., Zhao X., Lin Q. (2022). Towards detection of biomarkers in the eye using an aptamer-based graphene affinity nanobiosensor. Talanta.

[37] Amezcua-Castillo E., González-Pacheco H., Martín A.S.-S., Méndez-Ocampo P., Gutierrez-Moctezuma I., Massó F., Sierra-Lara D., Springall R., Rodríguez E., Arias-Mendoza A. (2023). C-Reactive Protein: The quintessential marker of systemic inflammation in coronary artery disease—Advancing toward precision medicine. Biomedicines.

[38] Luan Y.-Y., Yao Y.-M. (2018). The clinical significance and potential role of C-reactive protein in chronic inflammatory and neurodegenerative diseases. Front. Immunol..

[39] Kruse J., Wörner J., Schneider J., Dörksen H., Pein-Hackelbusch M. (2024). Methods for estimating the detection and quantification limits of key substances in beer maturation with electronic noses. Sensors.

[40] Gegenschatz S.A., Chiappini F.A., Teglia C.M., de la Peña A.M., Goicoechea H.C. (2022). A tutorial for computing limits of detection and quantification in univariate calibration for complex samples. Anal. Chim. Acta.

[41] Chen J.-H., Jang C., Adam S., Fuhrer M.S., Williams E.D., Ishigami M. (2008). Charged-impurity scattering in graphene. Nat. Phys..

[42] Dong X., Fu D., Fang W., Shi Y., Chen P., Li L. (2009). Doping Single-Layer Graphene with Aromatic Molecules. Small.

[43] Ansar W., Ghosh S. (2013). C-reactive protein and the biology of disease. Immunol. Res..

[44] Kunes P., Holubcova Z., Kolackova M., Krejsek J. (2012). Pentraxin 3 (PTX 3): An endogenous modulator of the inflammatory response. Mediat. Inflamm..

[45] Arimondo P.B. (2000). Interaction of human DNA topoisomerase I with G-quartet structures. Nucleic Acids Res..

[46] Valero C., Lee M., Hoen D., Weiss K., Kelly D.W., Adusumilli P.S., Paik P.K., Plitas G., Ladanyi M., Postow M.A. (2021). Pretreatment neutrophil-to-lymphocyte ratio and mutational burden as biomarkers of tumor response to immune checkpoint inhibitors. Nat. Commun..

[47] Dong X., Shi Y., Huang W., Chen P., Li L. (2010). Electrical Detection of DNA Hybridization with Single-Base Specificity Using Transistors Based on CVD-Grown Graphene Sheets. Adv. Mater..

[48] Fu L., Zheng Y., Li X., Liu X., Lin C.-T., Karimi-Maleh H. (2023). Strategies and applications of graphene and its derivatives-based electrochemical sensors in cancer diagnosis. Molecules.

[49] Blümmel J., Perschmann N., Aydin D., Drinjakovic J., Surrey T., Lopez-Garcia M., Kessler H., Spatz J.P. (2007). Protein repellent properties of covalently attached PEG coatings on nanostructured SiO2-based interfaces. Biomaterials.

[50] Fernandez-Villamarin M., Sousa-Herves A., Correa J., Munoz E.M., Taboada P., Riguera R., Fernandez-Megia E. (2016). The effect of PEGylation on multivalent binding: A surface plasmon resonance and isothermal titration calorimetry study with structurally diverse PEG-dendritic GATG copolymers. ChemNanoMat.

[51] Malmsten M., Emoto K., Van Alstine J.M. (1998). Effect of chain density on inhibition of protein adsorption by poly(ethylene glycol) based coatings. J. Colloid Interface Sci..

[52] Ping J., Zhou Y., Wu Y., Papper V., Boujday S., Marks R.S., Steele T.W.J. (2015). Recent advances in aptasensors based on graphene and graphene-like nanomaterials. Biosens. Bioelectron..

[53] Wang Z., Dai W., Zhang Z., Wang H. (2025). Aptamer-based graphene field-effect transistor biosensor for cytokine detection in undiluted physiological media for cervical carcinoma diagnosis. Biosensors.

[54] Arya S.K., Estrela P. (2020). Electrochemical ELISA Protein Biosensing in Undiluted Serum Using a Polypyrrole-Based Platform. Sensors.

[55] Tsai M.-Z., Hsiung C.-T., Chen Y., Huang C.-S., Hsu H.-Y., Hsieh P.-Y. (2018). Real-time CRP detection from whole blood using micropost-embedded microfluidic chip incorporated with label-free biosensor. Analyst.

[56] Raj V., Hari P.R., Antony M., Sreenivasan K. (2010). Selective estimation of C-reactive protein in serum using polymeric formulations without antibody. Sens. Actuators B Chem..

[57] Vikholm-Lundin I., Albers W.M. (2006). Site-directed immobilisation of antibody fragments for detection of C-reactive protein. Biosens. Bioelectron..

