# Supplementary material for: Optimizing Surface Functionalization for Aptameric Graphene Nanosensors in Undiluted Physiological Media
Source: Sensors (Basel). 2026 Jan 22;26(2):744. doi: 10.3390/s26020744 (PMC12846176; doi:10.3390/s26020744)
Supplement: Supplementary file 1 [file sensors-26-00744-s001.zip › sensors-4072855-supplementary.pdf]

Supporting Information

Table of contents

Figure S1. Specificity of nanosensors with a parallel surface modification scheme.

Figure S2. Transfer characteristic curves in response to CRP for PEGylated nanosensors with different surface modification schemes.

Figure S3. Transfer characteristic curves in response to CRP for PEGylated nanosensors modified with PEG at different molecular weights.

Figure S4. Transfer characteristic curves in response to CRP for PEGylated nanosensors with different PEG surface densities.

Figure S5. Transfer characteristic curves in response to CRP for PEGylated nanosensors with different aptamer surface densities.

1. Nanosensor Design

The nanosensor is an electrolyte-gated field-effect transistor (FET) that uses a graphene conducting channel between the source and drain electrodes. The graphene is modified with PEG to minimize nonspecific binding and a nucleic acid aptamer specific to the target biomarker. The aptamer specifically binds to the target biomarker, thereby inducing a change in the carrier concentration in the graphene and hence in the drain–source current of the nanosensor, which is measured to determine the concentration of the biomarker [S1]. PEG, which has been reported to increase the Debye screening length in conditioned media of high ionic strength [S2], reduces nonspecific adsorption of background molecules in physiological media.

2. Nanosensor Fabrication

A Si wafer coated with a 285 nm layer of SiO2 was used as a substrate. A 5 nm Ti layer and 50 nm Au layer were deposited by E-beam evaporation and patterned by photolithography on the wafer to form the source, drain, and gate electrodes. The wafer was then sonicated with acetone to remove the photoresist and cleaned by rinsing with isopropyl alcohol and deionized water. The wafer was finally dried on a hot plate at 150 °C and diced. Finally, chemical vapor deposition (CVD) with synthesized graphene was transferred onto electrodes and patterned to define the sensing channel through photolithography and oxygen plasma etching.

3. Nanosensor Functionalization

Two surface modification schemes were implemented. In one scheme (serial attachment), the aptamer was attached to PEG, which was in turn attached to the graphene surface; in the other scheme (parallel attachment), the aptamer and PEG were both directly attached to the graphene surface.

Serial surface modification scheme. To immobilize PEG onto the graphene surface via serial attachment, the nanosensor was first modified with 10 mM PASE, which served as a linker through π-π stacking interactions [S2]. The nanosensor chip was immersed in PASE solution overnight at room temperature for PASE attachment. After removal of free PASE by sequential rinses with dimethylformamide (DMF) and phosphate-buffered saline (PBS), the chip was incubated with PEG solution, again overnight at room temperature, for attachment of PEG to the

surface. A mixture of 1-ethyl-3-(3'-dimethylaminopropyl) carbodiimide hydrochloride (EDC·HCl) (0.1 M) and N-hydroxysulfosuccinimide (NHS) (0.05 M) in PBS solution (pH=6) was used to activate the carboxylic group at the free end of PEG for 1 hour. The chip was then immersed in PBS-based aptamer solution at room temperature for the aptamer to be chemically attached to PEG via a condensation reaction between amnio and carboxylic groups. After rinsing with PBS, 100 mM ethanolamine was added onto the graphene surface for 1 hour for surface passivation, in which unreacted groups remaining on the graphene surface were deactivated or blocked [S2].

Parallel surface modification scheme. In the parallel attachment scheme for surface modification, PASE was attached to the graphene surface and used as a linker to immobilize both the aptamer and PEG. The aptamer anchored on the graphene surface formed a parallel configuration with respect to PEG. After modification, the unreacted PASE was passivated by ethanolamine.

4. Materials and Equipment

Chemical vapor deposition (CVD) graphene was purchased from Graphenea Inc. (Cambridge, MA). Human CRP and Pentraxin 3 (PTX 3) proteins were purchased from R&D Systems (Minneapolis, MN). Human serum, 1-pyrenebutanoic acid succinimidyl ester (PASE), and dimethylformamide (DMF) were purchased from Sigma-Aldrich (St. Louis, MO). PEG polymers were purchased from Creative PEGWorks (Chapel Hill, NC). A CRP-specific aptamer (5'-GGC AGG AAG ACA AAC ACG ATG GGG GGG TAT GAT TTG ATG TGG TTG TTG CAT GAT CGT GGT CTG TGG TGC TGT-3') was synthesized and purified by Integrated DNA Technologies (Coralville, IA).

Raman spectroscopy was performed with a Renishaw inVia Raman microscope (UK) with 532 nm excitation. Atomic force microscopy (AFM) was performed with a Bruker Dimension Icon AFM. Energy-dispersive X-ray spectroscopy (EDS) was integrated with the SEM system (Oxford Instruments EDS detector). A Keithley 4200 Semiconductor Parameter Analyzer and a Ag/AgCl reference electrode (BASi Research Products) were used for electrical characterizations and sensor measurements.

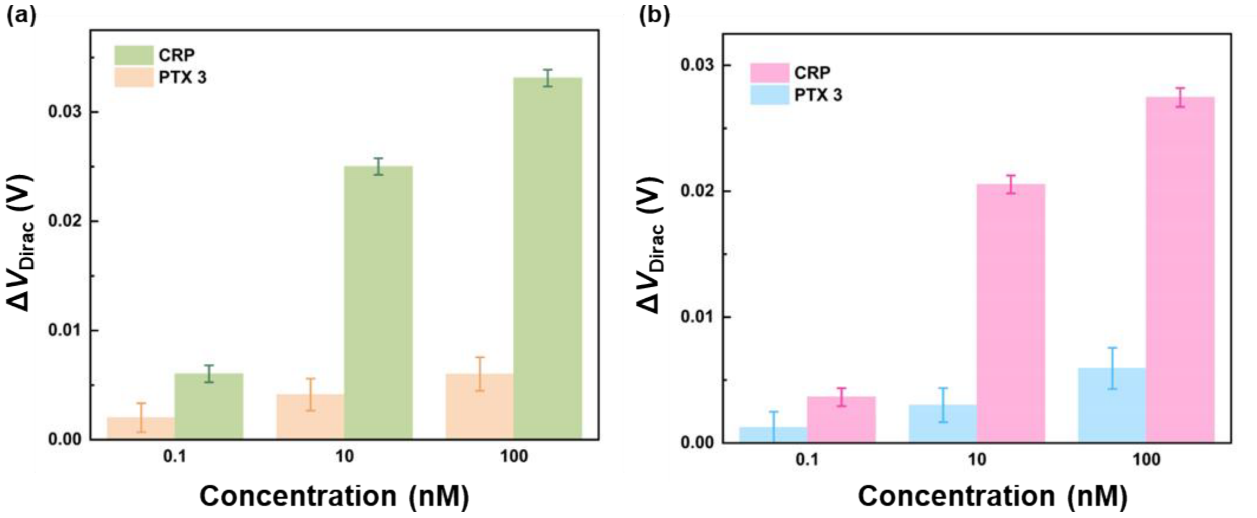

Figure S1. The sensing response of the PEGylated nanosensor with a parallel modification scheme to CRP and control protein (PTX 3) in **(a)** PBS and **(b)** human serum, respectively.

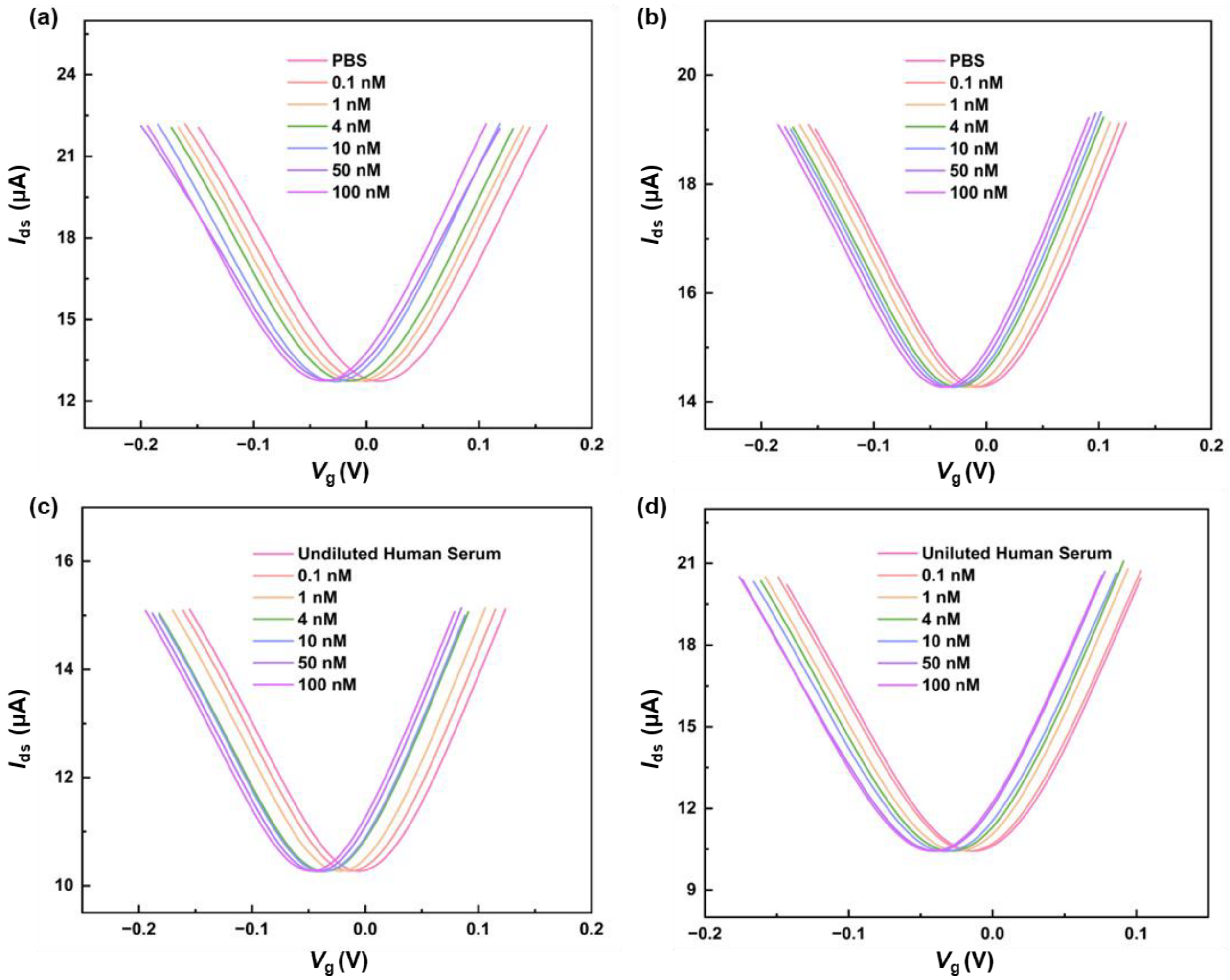

Figure S2. Responses of the nanosensors with different surface modification schemes. Transfer characteristic curves in response to CRP for the PEGylated nanosensors with **(a)** serial and **(b)** parallel surface modification schemes in PBS. Transfer characteristics curves in response to CRP for the PEGylated nanosensors with **(c)** serial and **(d)** parallel surface modification schemes in human serum.

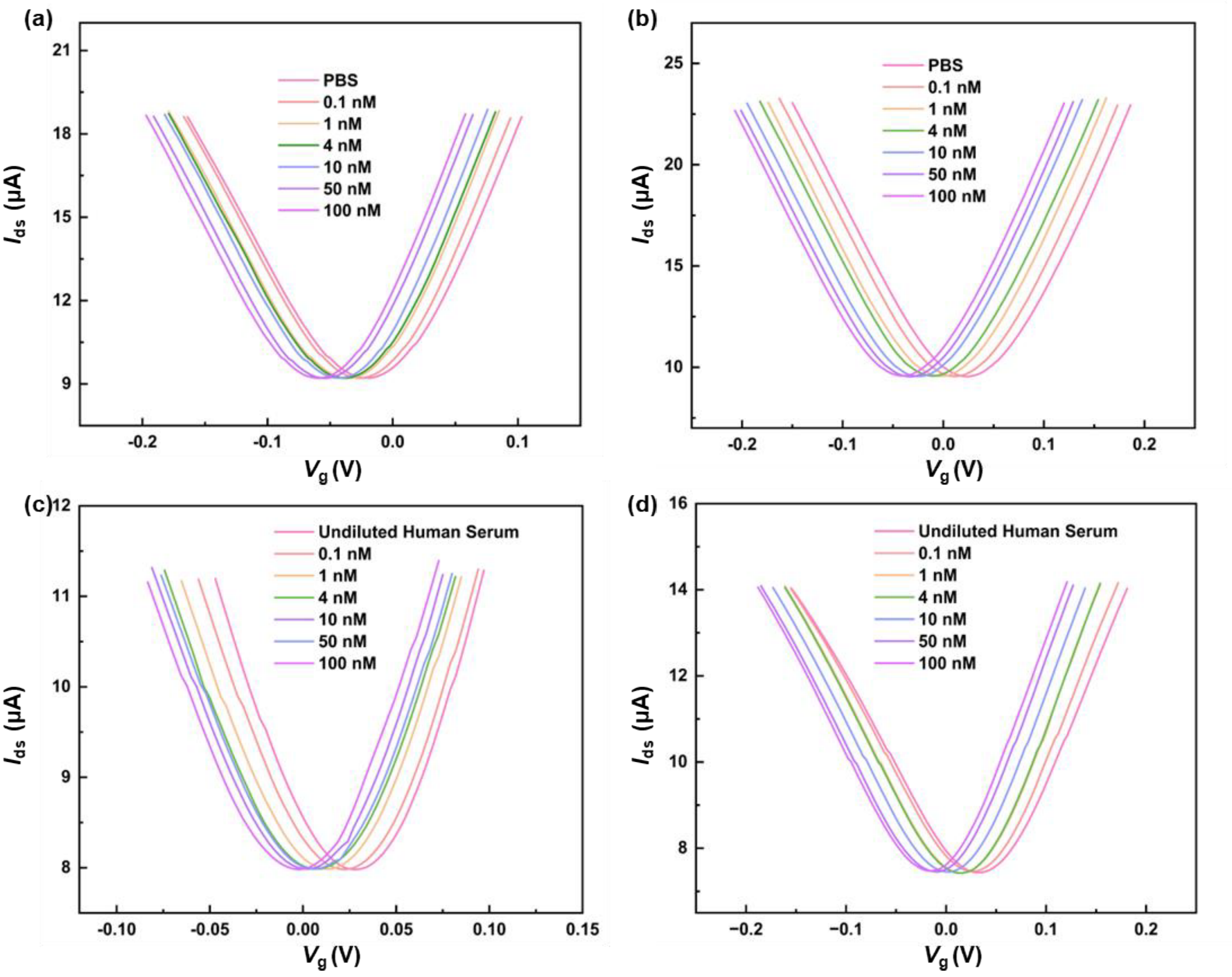

Figure S3. Responses of the nanosensors modified with PEG of different molecular weights. Transfer characteristic curves in response to CRP for the PEGylated nanosensors modified with PEG of molecular weights **(a)** 1000 and **(b)** 5000 Da in PBS. Transfer characteristics curves in response to CRP for the PEGylated devices modified with PEG of molecular weights **(c)** 1000 and **(d)** 5000 Da in undiluted human serum.

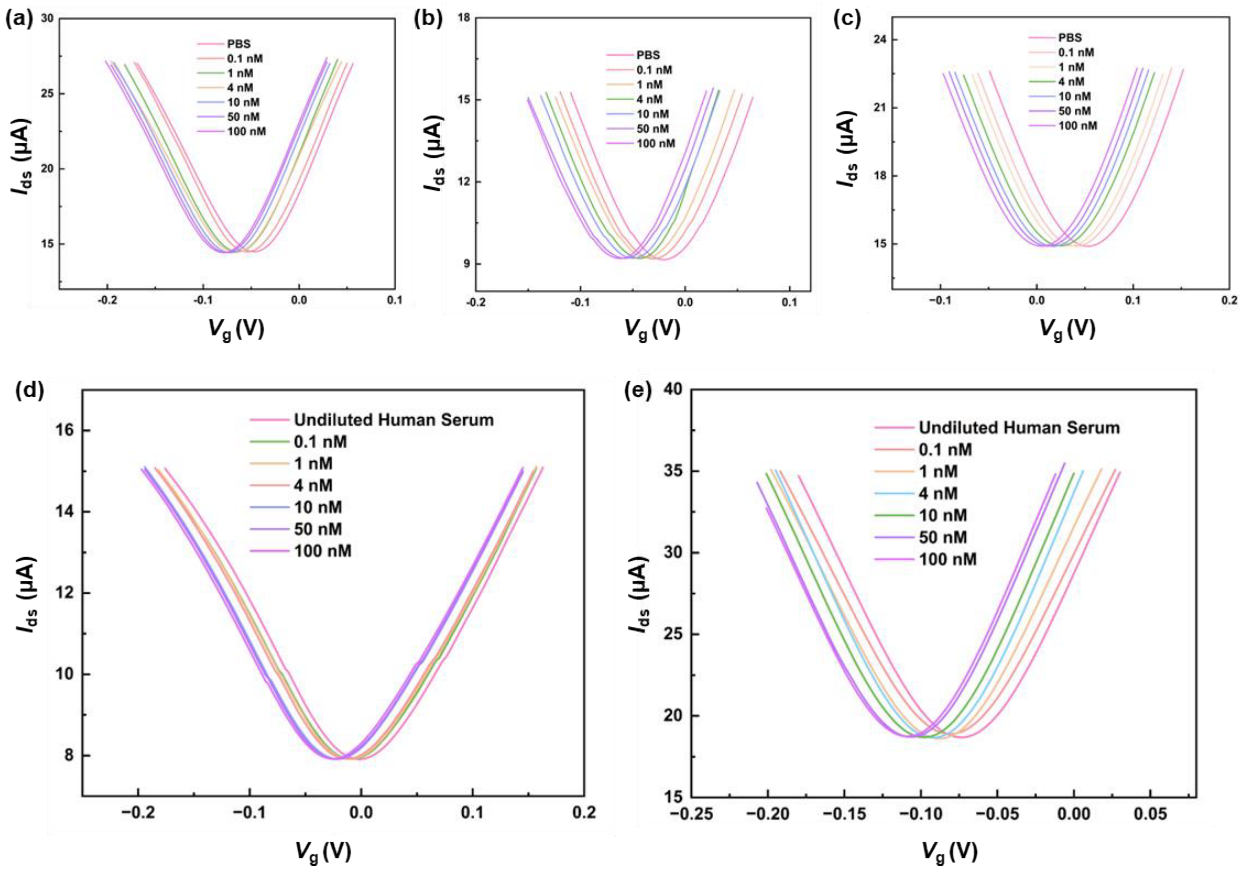

Figure S4. Responses of the nanosensors with different PEG surface densities. Transfer characteristic curves in response to CRP for the PEGylated devices modified with PEG of concentrations at **(a)** 1, **(b)** 5, and **(c)** 20 mM in PBS. Transfer characteristics curves in response to CRP for the PEGylated devices modified with PEG of concentrations at **(d)** 1 and **(e)** 5 mM in undiluted human serum.

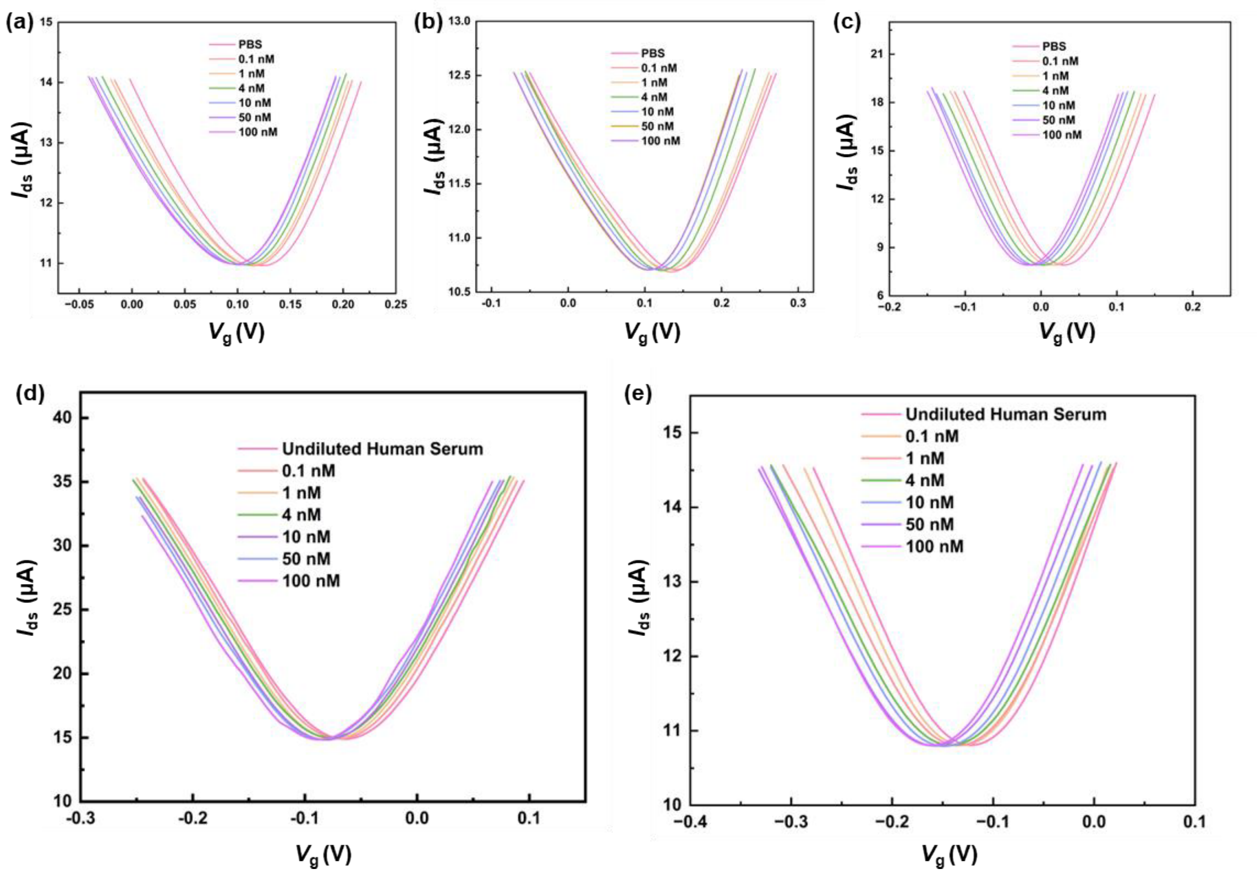

Figure S5. Responses of the nanosensors with different aptamer surface densities. Transfer characteristic curves in response to CRP for the PEGylated nanosensors modified with the aptamer at the concentrations of **(a)** 50, **(b)** 150, and **(c)** 1000 nM in PBS. Transfer characteristics curves in response to CRP for the PEGylated devices modified with the aptamer at the concentrations of **(d)** 50 and **(e)** 150 nM in undiluted human serum.

References

[S1] A. Béraud, M. Dahlin, B. Liedberg, Graphene field-effect transistors as bioanalytical sensors: design, operation and performance, Analyst 146 (2021) 403–428. https://doi.org/10.1039/D0AN01661F.

[S2] Z. Wang, W. Dai, Z. Zhang, H. Wang, Aptamer-based graphene field-effect transistor biosensor for cytokine detection in undiluted physiological media for cervical carcinoma diagnosis, Biosensors 15 (2025) 138. https://doi.org/10.3390/bios15030138.
